# Genetic signatures for *Helicobacter pylori* strains of West African origin

**DOI:** 10.1371/journal.pone.0188804

**Published:** 2017-11-29

**Authors:** Kennady K. Bullock, Carrie L. Shaffer, Andrew W. Brooks, Ousman Secka, Mark H. Forsyth, Mark S. McClain, Timothy L. Cover

**Affiliations:** 1 Department of Medicine, Vanderbilt University School of Medicine, Nashville, Tennessee, United States of America; 2 Department of Veterinary Science, University of Kentucky, Lexington, Kentucky, United States of America; 3 Vanderbilt Genetics Institute, Vanderbilt University School of Medicine, Nashville, Tennessee, United States of America; 4 Medical Research Council Unit The Gambia, Banjul, The Gambia; 5 Department of Biology, The College of William and Mary, Williamsburg, Virginia, United States of America; 6 Department of Pathology, Microbiology and Immunology, Vanderbilt University School of Medicine, Nashville, Tennessee, United States of America; 7 Veterans Affairs Tennessee Valley Healthcare System, Nashville, Tennessee, United States of America; National Cancer Center, JAPAN

## Abstract

*Helicobacter pylori* is a genetically diverse bacterial species that colonizes the stomach in about half of the human population. Most persons colonized by *H*. *pylori* remain asymptomatic, but the presence of this organism is a risk factor for gastric cancer. Multiple populations and subpopulations of *H*. *pylori* with distinct geographic distributions are recognized. Genetic differences among these populations might be a factor underlying geographic variation in gastric cancer incidence. Relatively little is known about the genomic features of African *H*. *pylori* strains compared to other populations of strains. In this study, we first analyzed the genomes of *H*. *pylori* strains from seven globally distributed populations or subpopulations and identified encoded proteins that exhibited the highest levels of sequence divergence. These included secreted proteins, an LPS glycosyltransferase, fucosyltransferases, proteins involved in molybdopterin biosynthesis, and Clp protease adaptor (ClpS). Among proteins encoded by the *cag* pathogenicity island, CagA and CagQ exhibited the highest levels of sequence diversity. We then identified proteins in strains of Western African origin (classified as hspWAfrica by MLST analysis) with sequences that were highly divergent compared to those in other populations of strains. These included ATP-dependent Clp protease, ClpS, and proteins of unknown function. Three of the divergent proteins sequences identified in West African strains were characterized by distinct insertions or deletions up to 8 amino acids in length. These polymorphisms in rapidly evolving proteins represent robust genetic signatures for *H*. *pylori* strains of West African origin.

## Introduction

*Helicobacter pylori* is a Gram-negative bacterial species that persistently colonizes the stomach in about half of the world’s human population. *H*. *pylori* has a high mutation rate, and strains from unrelated persons exhibit a high level of genetic diversity [1–4]. *H*. *pylori* strains from various geographic areas can be classified into distinct populations and subpopulations, based on multi-locus sequence typing (MLST) analysis [5–7]. *H*. *pylori* genetic diversity decreases with increasing geographic distance from Africa, the origin of *Homo sapiens* [6]. Therefore, *H*. *pylori* is thought to have co-evolved with humans over the past 100,000 years [8], and geographic differences among *H*. *pylori* strains reflect ancient human migration events [5, 6, 9].

Despite *H*. *pylori’s* long evolutionary history with humans, *H*. *pylori* colonization is a risk factor for the development of non-cardia gastric cancer and peptic ulcer disease. In 1994, the International Agency for Research on Cancer classified *H*. *pylori* as a bacterial carcinogen [10]. Genetic variation among *H*. *pylori* strains is known to be an important factor influencing the outcome of infection [1, 11]. For example, strains that contain the *cag* pathogenicity island (PAI), which encodes CagA (a secreted effector protein) and a type IV secretion system [12–15], are associated with a higher risk of gastric cancer or peptic ulceration compared to strains that do not contain the *cag* PAI [1, 11]. Similarly, strains that produce active forms of the VacA toxin and strains that produce specific outer membrane proteins have been linked to an increased risk of gastric cancer or peptic ulceration [11, 16].

The prevalence of *H*. *pylori* infection and the incidence of gastric cancer each vary geographically [17, 18]. In general, developing countries have a higher prevalence of *H*. *pylori* infection than developed countries. East Asia and certain parts of South America and Central America have high rates of gastric cancer, and several African countries have a relatively low rate of gastric cancer [18, 19]. The age-adjusted gastric cancer incidence rate is about 10-fold higher in males from East Asia than in West African males [18]. Interestingly, many African countries have a relatively low incidence of gastric cancer despite a high prevalence of *H*. *pylori*. Holcombe and colleagues termed this phenomenon “the African enigma”, and suggested that African strains of *H*. *pylori* may have reduced virulence [20].

A low rate of gastric cancer in Africa could be due to co-evolution of African strains with African humans over a very long period of time, allowing the microbe and host to form a more harmonious relationship [21, 22]. Consistent with the low rate of gastric cancer in many parts of Africa, studies in Colombia detected a reduced incidence of premalignant gastric lesions in humans of African ancestry who were colonized with *H*. *pylori* strains of African origin, compared to other populations residing in Colombia [21, 22]. Conversely, *H*. *pylori* strains of African origin were associated with pre-neoplastic gastric pathology in humans of Amerindian origin [21, 22]. These observations suggest that prolonged *H*. *pylori*-human coevolution is associated with attenuation of gastric pathology, whereas admixture of *H*. *pylori* strains and humans of different geographic origins can potentially lead to adverse outcomes.

Several other factors could contribute to a relatively low incidence of gastric cancer in Africa, including a high rate of intestinal parasitic infections that might attenuate *H*. *pylori* virulence [23], possible protective effects associated with simultaneous colonization by *cagA*-positive and *cagA*-negative strains [24], composition of the gastric or intestinal microbiome, or composition of the diet [25, 26]. A low reported incidence of gastric cancer might also reflect limitations in the availability of diagnostic procedures (such as endoscopy) and incomplete reporting [27].

Geographic differences in *H*. *pylori* virulence might be attributable to geographic variation in the presence of strain-specific (non-conserved) bacterial genes, or alternatively, might be due to geographic variation in *H*. *pylori* protein sequences that are associated with differences in protein function. For example, there is a high level of geographic variation in sequences of *H*. *pylori* CagA [12, 28–30], and the CagA proteins produced by East Asian strains cause more extensive alterations in gastric epithelial cells than CagA proteins produced by strains from other parts of the world [12, 30–33].

Thus far there has been relatively little effort to determine how African *H*. *pylori* strains differ from other populations of strains. MLST analysis is a useful approach for identifying *H*. *pylori* strains of African origin, but most of the polymorphisms analyzed by MLST are synonymous substitutions in housekeeping genes, which are unlikely to be associated with alterations in protein function. Moreover, the individual substitution mutations in housekeeping genes are not robust markers of African ancestry. One previous study reported that a 180-bp insertion in an intergenic region was present more frequently in strains of West African origin than in strains of European origin [34]. Thus far, this 180-bp insertion is the only genetic marker that can be used independently (i.e. not as a part of an MLST panel) for recognition of African strains. Therefore, the goal of the current study was to systematically analyze sequence diversity among *H*. *pylori* strains from diverse geographic origins, and identify proteins that have undergone a high level of sequence divergence in strains of West African origin.

## Results

### Identification of highly divergent proteins in geographically dispersed *H*. *pylori* populations

As a first approach for identifying *H*. *pylori* proteins that have undergone high levels of sequence diversification, we undertook a comparative genomic analysis of representative strains from globally distributed *H*. *pylori* populations. We analyzed seven strains that had previously been classified into seven distinct populations or subpopulations, based on MLST analysis (Table 1). Characteristics of the strains are shown in Table 1.

**Table 1:** Characteristics of *H*. *pylori* strains from geographically dispersed populations.

Strain	MLST classification	Geographic Source	Disease State	*cagA*^a^	Genome size (Mb)	Jhp0153-jhp0152 180-bp insertion^b^
26695	hpEurope	United Kingdom	Gastritis	+	1.67	-
CC33C	hspSAfrica	South Africa	Not known	+	1.66	NA^c^
F30	hspEAsia	Unknown	Not known	+	1.58	-
India7	hpAsia2	India	Not known	+	1.68	-
J99	hspWAfrica	USA	Duodenal ulcer	+	1.64	+
Shi470	hspAmerind	Peru	Gastric ulcer	+	1.61	-
SouthAfrica7	hpAfrica2	SouthAfrica	Not known	-	1.68	-

^a^ Presence or absence of the *cagA* gene in the indicated genome.

^b^ Presence or absence of a 180-bp insertion, located in the intergenic region between JHP0153 and JHP1052 in strain J99, in the indicated genomes.

^c^NA, not applicable. Presence or absence of this insertion in strain CC33C could not be ascertained based on analysis of the genome sequence deposited in Genbank.

Comparative analysis of the seven strains with nWayComp identified 1187 gene products encoded by all seven strains. The mean ± SD amino acid sequence identity of orthologous protein sequences among the seven strains was 94.2 ± 0.06%. Seventy-two of the 1187 proteins exhibited a high level of sequence diversity (S1 Table), based on the criteria described in Methods. In comparisons of orthologous protein sequences among the seven strains, each of the divergent proteins had a mean amino acid sequence identity of <90%. Representative examples of the divergent proteins are shown in Table 2. For comparison, examples of proteins involved in transcription and translation and retaining highly conserved sequences are shown in S2 Table (amino acid sequence identities ≥98.0%). The list of divergent proteins includes secreted proteins [35], a lipopolysaccharide glycosyltransferase, and fucosyltransferases (Table 2 and S1 Table). Two of the divergent proteins (encoded by HP0800 and HP0769) are predicted to be involved in synthesis of molybdopterin (a cofactor present in most molybdenum-containing enzymes). Two others [the LPS 1,2-glycosyltransferase encoded by HP0159 and the protein encoded by HP1029] were identified as critical factors required for activity of the *cag* type IV secretion system [36]. The functions of many of the other divergent proteins listed in S1 Table are not known.

**Table 2:** Examples of proteins that exhibit high levels of sequence diversity among geographically dispersed populations of *H*. *pylori*.

Gene no. (strain 26695)	Gene no. (strain J99)	Mean % amino acid identity^a^	Annotation or predicted function	Protein Length (amino acids)^b^
HP0032	JHP0028	80.0	ATP-dependent Clp protease adaptor protein	91
HP0159	JHP0147	88.2	LPS 1,2 glycosyltransferase	372
HP0160	JHP0148	88.7	HcpD penicillin-binding protein	306
HP0379	JHP1002	76.8	Alpha 1,3 fucosyltransferase	425
HP0492	JHP0444	76.3	Neuraminylactose-binding hemagglutinin paralog of HpaA	278
HP0651	JHP0596	77.6	Alpha 1,3 fucosyltransferase	476
HP0769	JHP0706	87.5	Molybdopterin guanine dinucleotide biosynthesis protein A	201
HP0800	JHP0736	88.5	Molybdopterin converting factor subunit 2	145
HP0907	JHP0843	86.6	Predicted hook assembly protein	301
HP1051	JHP0374	87.1	Predicted coding region	140
HP1053	JHP0372	87.9	Septum site-directing protein MinC	217
HP1286	JHP1206	86.4	Predicted secreted protein	182
HP1551	JHP1448	79.9	Predicted secreted protein	127

^a^ Based on comparisons among 7 globally dispersed strains of *H*. *pylori* (Table 1)

^b^Length of protein encoded by reference strain 26695

In a previous study, we identified proteins with sequences that were highly divergent in East Asian *H*. *pylori* strains compared to non-East Asian strains [37]. Among the 72 divergent proteins identified in the current analysis of globally dispersed strains, 22 were also identified in the previous study of East Asian strains [37] (S1 Table). The substantial concordance in results of these two analyses bolsters the conclusion that these are rapidly evolving proteins.

### Comparative analysis of proteins encoded by the *cag* PAI

The foregoing analyses focused on proteins encoded by all of the *H*. *pylori* strains selected for study, and therefore, these analyses did not consider genetic variation in proteins encoded by the *cag* PAI, which is present in some strains but not others. Previous studies have shown that there is a high level of sequence variation among CagA proteins produced by different populations of *H*. *pylori* strains [12, 28, 29]. For example, the sequences of CagA proteins produced by East Asian or Amerindian strains of *H*. *pylori* are highly divergent compared to sequences of CagA produced by European strains [12, 28–30, 37–39], and this sequence variation is associated with different activities of the corresponding CagA proteins within host cells [12, 30, 31]. To systematically analyze sequence diversity in proteins encoded by the *cag* PAI, we analyzed the six *cagA*-positive strains from geographically dispersed regions (Table 1). Twenty-two intact *cag* PAI gene sequences were present in all six strains. The apparent lack of an intact *cagY* sequence in one or more of these strains is probably attributable to challenges with the sequencing of this gene, due to numerous repeat elements. The average amino acid sequence identity for the 22 Cag proteins among the six *H*. *pylori* strains was 95.0% (Table 3). As expected, CagA exhibited the highest level of sequence diversity (mean amino acid sequence identity of 81.6%). CagQ, a protein of unknown function, also exhibited a relatively high level of sequence diversity (84.2%) compared to other Cag proteins. These findings are similar to the results reported in a previous study, which analyzed the *cag* PAI in a large collection of strains and detected the highest levels of sequence diversity in CagA, followed by CagQ [28].

**Table 3:** Sequence diversity among *cag* pathogenicity island proteins in strains from geographically dispersed populations.

Gene number (strain 26695)	Mean % amino acid identity^a^	*cag* PAI protein
HP0520	95.3	Cag1
HP0522	94.7	Cag3
HP0523	93.0	Cag4
HP0524	98.5	Cag5
HP0526	97.6	CagZ
HP0529	98.2	CagW
HP0530	98.3	CagV
HP0531	96.5	CagU
HP0532	98.6	CagT
HP0534	96.3	CagS
HP0535	84.2	CagQ
HP0536	96.0	CagP
HP0537	98.4	CagM
HP0538	95.3	CagN
HP0539	96.0	CagL
HP0540	95.7	CagI
HP0541	92.8	CagH
HP0542	96.9	CagG
HP0543	96.8	CagF
HP0545	95.7	CagD
HP0546	94.2	CagC
HP0547	81.6	CagA

^a^ Based on comparisons among globally dispersed strains of *H*. *pylori* (Table 1)

### Genetic signatures for West African *H*. *pylori* strains

The proteins listed in Table 2 and S1 Table exhibit a high level of sequence diversity when comparing *H*. *pylori* strains from disparate global populations. We hypothesize that the protein sequences in the seven globally dispersed *H*. *pylori* strains selected for analysis might be generally representative of the sequences found in those seven populations of strains. To test whether the sequence diversity detected among the seven global *H*. *pylori* strains could be used as a tool for distinguishing among different populations of strains, we focused the next analysis on strains of West African origin, a population that has thus far not been studied in much detail. We selected eight strains of West African origin (S3 Table), as described in Methods, including reference strain J99 (hspWAfrica), which was used in the previous analysis (Table 1). For comparison, we selected 8 strains of European origin (hpEurope) (S3 Table), including reference strain 26695, which was also used in the previous analysis (Table 1). MLST analysis confirmed the classification of the two groups of strains as hspWAfrica and hpEurope, respectively (S1 Fig). Additional characteristics of the strains are shown in S3 Table.

A previous study reported the existence of a 180-bp insertion in an intergenic region of reference strain J99 (between genes JHP0152 and JHP0153) and other strains of African origin, which was absent from most strains of non-African origin [34]. This insertion was present in all 8 of the hspWAfrica strains, as well as in one hpEurope strain (SJM180), but was absent from the other hpEurope strains (S3 Table). For comparison, among the global set of *H*. *pylori* strains (Table 1), the 180-bp insertion in the JHP0153-JHP0152 intergenic region was absent from all of the strains except for J99 (hspWAfrica). These results support the conclusion that this insertion in an intergenic region is a useful marker for strains of West African origin [34].

Comparative genomic analysis of the hspWAfrican and hpEurope strains using nWaycomp identified 1,113 proteins encoded by all 16 of the genomes. A large number of protein-encoding genes were present in only a subset of the 16 strains, but we did not identify any that were consistently present in hspWAfrica strains and absent from hpEurope strains, or vice versa. The mean amino acid identity in hpEurope-hspWAfrica comparisons for the full set of 1,113 orthologous proteins was 94.8%. Examples of proteins involved in transcription and translation and retaining highly conserved sequences are shown for comparison in S4 Table (≥98.5% amino acid identity in hpEurope-hspWAfrica comparisons). We identified eight proteins that were markedly divergent in hspWAfrica strains compared to hpEurope strains (Table 4), using the criteria described in Methods. In comparisons of protein sequences in the hspWAfrica strains with orthologous sequences in the hpEurope strains, the divergent proteins exhibited a mean amino acid sequence identity of 84.1% (range 73.3 to 89.9%).

**Table 4:** Proteins that exhibit high levels of sequence diversity when comparing hspWAfrica and hpEurope strains.

Gene number (strain 26695)	Mean % amino acid identity, hpEurope—hspWAfrica	Mean % amino acid identity, intra-hpEurope	Mean % amino acid identity, intra-hspWAfrica	Annotation or predicted function	Protein length (amino acids)^a^
HP0032	73.3	88.3	99.0	ATP-dependent Clp protease adaptor ClpS	91
HP0033	89.9	96.1	98.9	ATP-dependent Clp protease	741
HP0257	88.3	94.3	93.9	Predicted coding region	219
HP0384	87.9	94.0	97.7	SPOR domain-containing protein	250
HP0408	80.5	90.3	97.3	Predicted coding region	162
HP1051	87.5	93.5	95.6	Predicted coding region	140
HP1053	87.0	93.3	98.4	Septum site directing protein MinC	217
HP1070	78.5	87.4	88.8	Predicted coding region	84

^a^Length of protein encoded by reference strain 26695.

Notably, four of the divergent proteins are encoded by two pairs of genes localized in the same region of the chromosome (HP0032, HP0033, HP1051 and HP1053 in reference strain 26695; JHP0028, JHP0029, JHP0374 and HP0372 in reference strain J99), and are likely co-transcribed. HP0032 and HP033 are predicted to have related functions. HP0032 encodes an ATP-dependent Clp protease and HP0033 encodes a Clp protease adaptor (ClpS). ClpS modulates the specificity of protein degradation by the ClpAP chaperone-protease complex [40]. HP1053 is annotated as a septum site directing protein (MinC), and has a role in maintenance of *H*. *pylori* cell morphology [41]. Annotations or predicted functions of the other divergent proteins are shown in Table 4.

**Fig 1:**
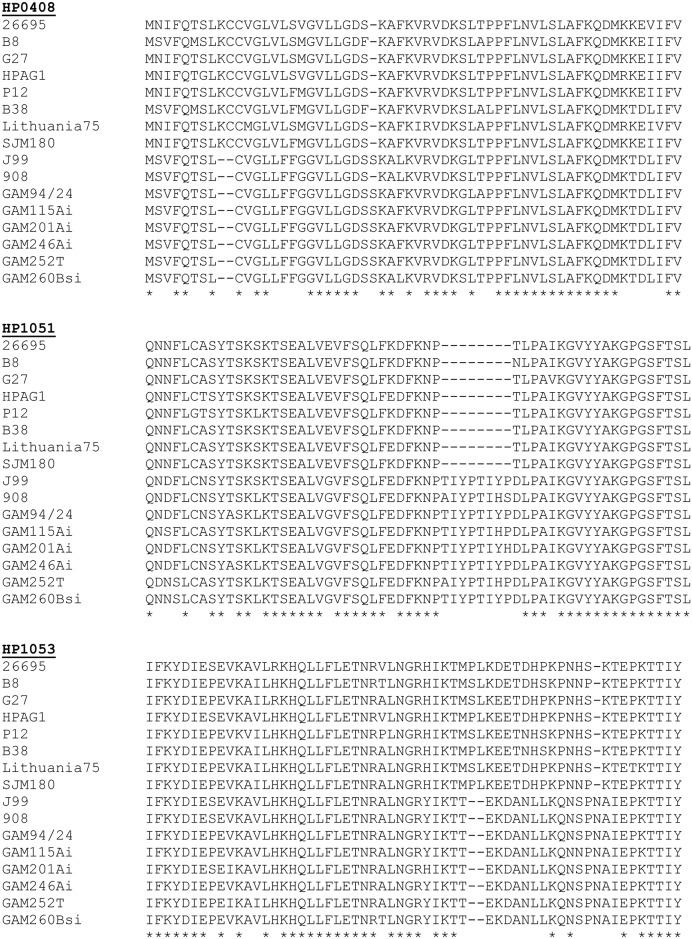
Sequence alignments of three proteins exhibiting high levels of sequence divergence when comparing hpEurope and hspWAfrica strains. A) Amino acid sequence alignment of proteins encoded by A) HP0408, B) HP1051, and C) HP1053. The HP0408 alignment corresponds to the amino-terminus, and the other two alignments correspond to internal sequences. For each protein, the first eight lines are sequences from hpEurope strains and the last eight lines are sequences from hspWAfrica strains.

Multiple sequence alignments for three of the proteins considered divergent when comparing hspWAfrica strains with hpEurope strains (HP0408, HP0151, and HP0153) are shown in Fig 1. These alignments illustrate the presence of distinct insertions or deletions, up to eight amino acids in length, as well as individual amino acid polymorphisms that are differentially present in hspWAfrica strains and hpEurope strains (Fig 1).

The list of divergent proteins in Table 4 was then compared to the list of 72 divergent proteins found in the worldwide, geographic analysis (S1 Table). Three of the 8 proteins that were divergent when comparing hspWAfrica strains with hpEurope strains (HP0032, HP1051, and HP1053) were also identified as highly divergent in the analysis of globally distributed strains (Tables 2 and 4 and S1 Table). One of these (HP0384) was identified in a previous analysis that focused on proteins with sequences that are highly divergent when comparing East Asian *H*. *pylori* strains with non-Asian strains (hpEurope or hspWAfrica) [37].

To evaluate whether increased sequence diversity among the eight divergent proteins resulted from evolutionary pressures, we analyzed the nucleotide sequences encoding these proteins using the McDonald-Kreitman test of positive diversifying selection [42]. This test compares ratios of synonymous-to-non-synonymous polymorphisms and divergence (Ps/Pn and Ds/Dn) for sets of genes from two populations. The use of this test allows us to analyze whether a set of genes from either the African or European *H*. *pylori* population exhibits a stronger signal of adaptive pressure through increased rates of non-synonymous fixation compared to the corresponding set of genes from the other *H*. *pylori* population. We found that two of the eight genes (HP0257 and HP1053) were under diversifying selection (Table 5). The Neutrality Index (NI) for both of these genes was low (<0.3), indicating high rates of fixation for non-synonymous polymorphisms.

**Table 5:** Signatures of positive selection in genes encoding proteins with divergent sequences.

Gene ID	*Dn*^a^	*Ds*^a^	*Pn*^a^	*Ps*^a^	*P* value	NI^b^	α-Value^c^
HP0032	0	0	26	42	None	None	None
HP0033	16.37	8.02	253	137	0.821	1.104	-0.104
HP0257	1	3.01	71	26	0.036	0.122	0.877
HP0384	13.82	14.22	67	58	0.679	0.841	0.158
HP0408	8.47	4.03	61	66	0.183	2.274	-1.274
HP1051	0	1	54	31	0.19	0	1
HP1053	6.21	9.12	78	33	0.02	0.288	0.711
HP1070	2.05	3.03	30	53	0.847	1.195	-0.195

^a^*P*, polymorphisms within the populations; *D*, divergence or fixed difference between populations; n, nonsynonymous; s, synonymous.

^b^Neutrality Index (NI) calculated as NI = (*Pn*/*Ps*)/(*Dn*/*Ds*)

^c^Proportion of adaptive substitutions estimated as 1 –NI.

A previous analysis did not reveal any substantial divergence in CagA sequences when comparing European and African *H*. *pylori* strains [29]. Therefore, we analyzed the set of hpEurope and hspWAfrica strains to detect possible divergence in proteins encoded by the *cag* PAI (including CagA). Among the 13 strains that contained a *cag* PAI (S3 Table), 22 *cag* PAI genes were present in all 13 strains. The average amino acid sequence identity for this group of 22 proteins among the 13 strains was 95.7% (Table 6). Consistent with expectations, the highest level of sequence variation was found in CagA, and CagQ also displayed a relatively high level of sequence variation compared to other Cag proteins (Table 6). Notably, this analysis did not detect substantial divergence of CagA when comparing European and African strains (i.e., the mean level of amino acid sequence identity when comparing European and African strains was not substantially different from what was calculated for in intra-Europe or intra-Africa analyses) (Table 6). Similarly, manual inspection of the aligned CagA sequences identified relatively few polymorphisms that were unequally distributed between European and West African strains. Thus, the CagA sequences in East Asian and Amerindian populations of *H*. *pylori* exhibit geographically distinct features [12, 29, 37], but a similar divergence is not readily apparent when comparing European and West African strains.

**Table 6:** Sequence diversity among *cag* pathogenicity island proteins from African and European strains.

Gene no. (strain 26695)	% amino acid identity, hp-Europe-hspWAfrica	% amino acid identity, intra-hpEurope	% amino acid identity, intra-hspWAfrica	*cag* PAI protein
HP0520	95.4	95.5	98.0	Cag1
HP0522	94.0	97.2	98.1	Cag3
HP0523	93.3	95.3	98.3	Cag4
HP0524	98.7	98.8	99.5	Cag5
HP0526	97.4	98.7	99.1	CagZ
HP0528	98.1	98.6	99.3	CagX
HP0530	98.3	99.1	99.2	CagV
HP0531	96.6	96.9	99.1	CagU
HP0532	97.7	99.2	98.8	CagT
HP0534	94.7	96.0	97.2	CagS
HP0535	90.7	91.3	95.6	CagQ
HP0536	96.1	95.1	99.7	CagP
HP0537	98.5	99.0	99.5	CagM
HP0538	94.6	95.5	97.8	CagN
HP0539	96.5	97.2	97.8	CagL
HP0540	96.6	98.4	97.2	CagI
HP0541	96.3	94.7	99.0	CagH
HP0542	97.4	98.9	99.4	CagG
HP0543	96.6	96.9	99.1	CagF
HP0545	96.7	98.0	98.2	CagD
HP0546	94.5	96.7	95.5	CagC
HP0547	87.3	87.2	90.8	CagA

### Genetic signatures of West African strains are rare in most non-African populations of strains

**Fig 2:**
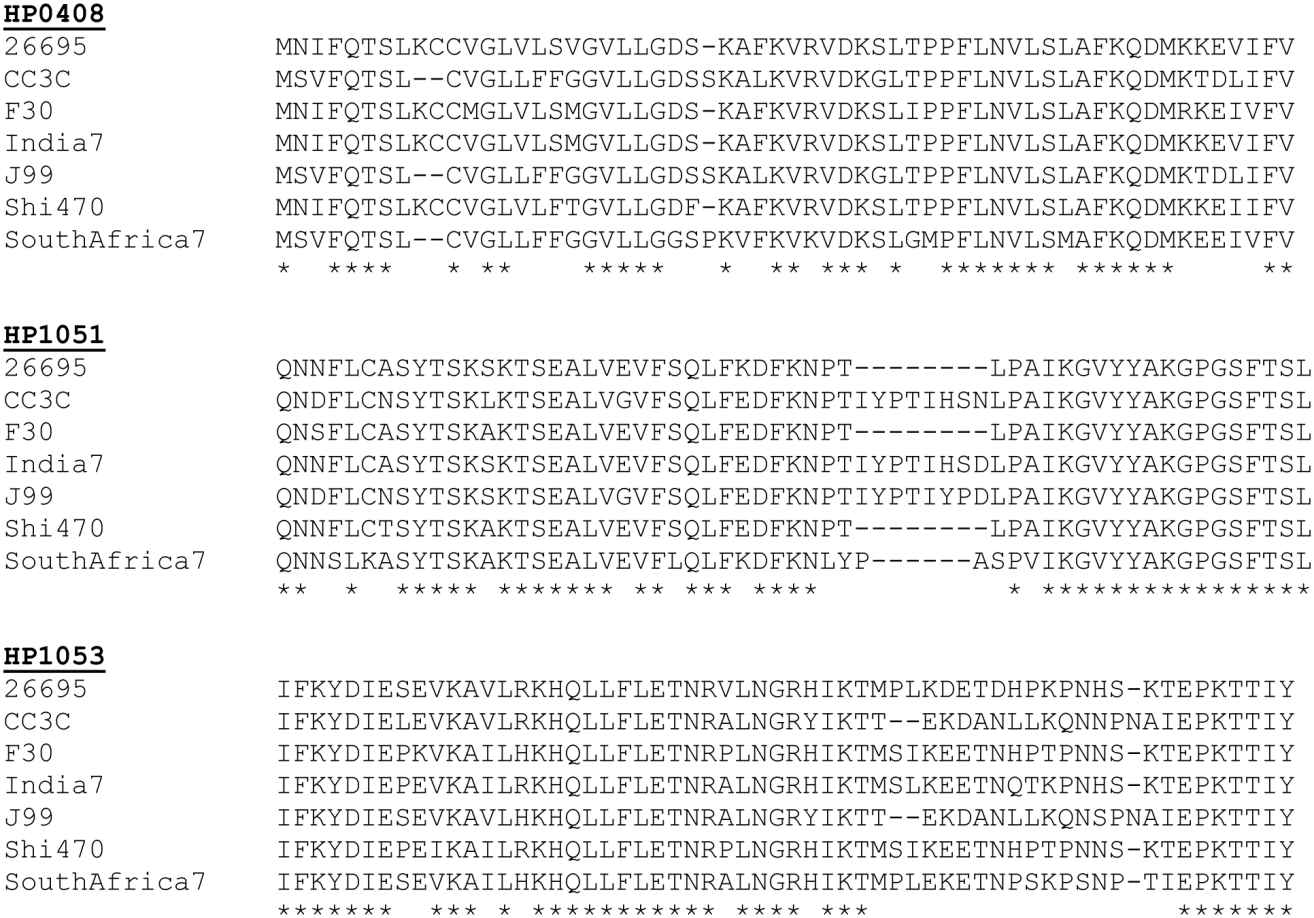
Sequence alignments of three proteins encoded by strains of diverse geographic origins. A) Amino acid sequence alignment of a portion of the translated gene region of A) HP0408, B) HP1051, and C) HP1053 in a comparison of seven, globally distributed strains. The HP0408 alignment corresponds to the amino-terminus, and the other two alignments correspond to internal sequences.

We next investigated whether the insertions and deletions identified in the comparison of hspWAfrica and hpEurope strains (in HP0408, HP0151, and HP0153, see Fig 1) were present in the set of globally distributed strains (Table 1). For both HP0408 and HP1053, the indel pattern characteristic of the hspWAfrica stains and exemplified by strain J99 was observed in strain CC3C (hspSAfrica), but not in any of the other strains. Similarly, for HP1051, the hspWAfrica pattern exemplified by strain J99 was observed in CC3C (hspSAfrica) and India7 (hpAsia2), but not in other strains (Fig 2).

**Fig 3:**
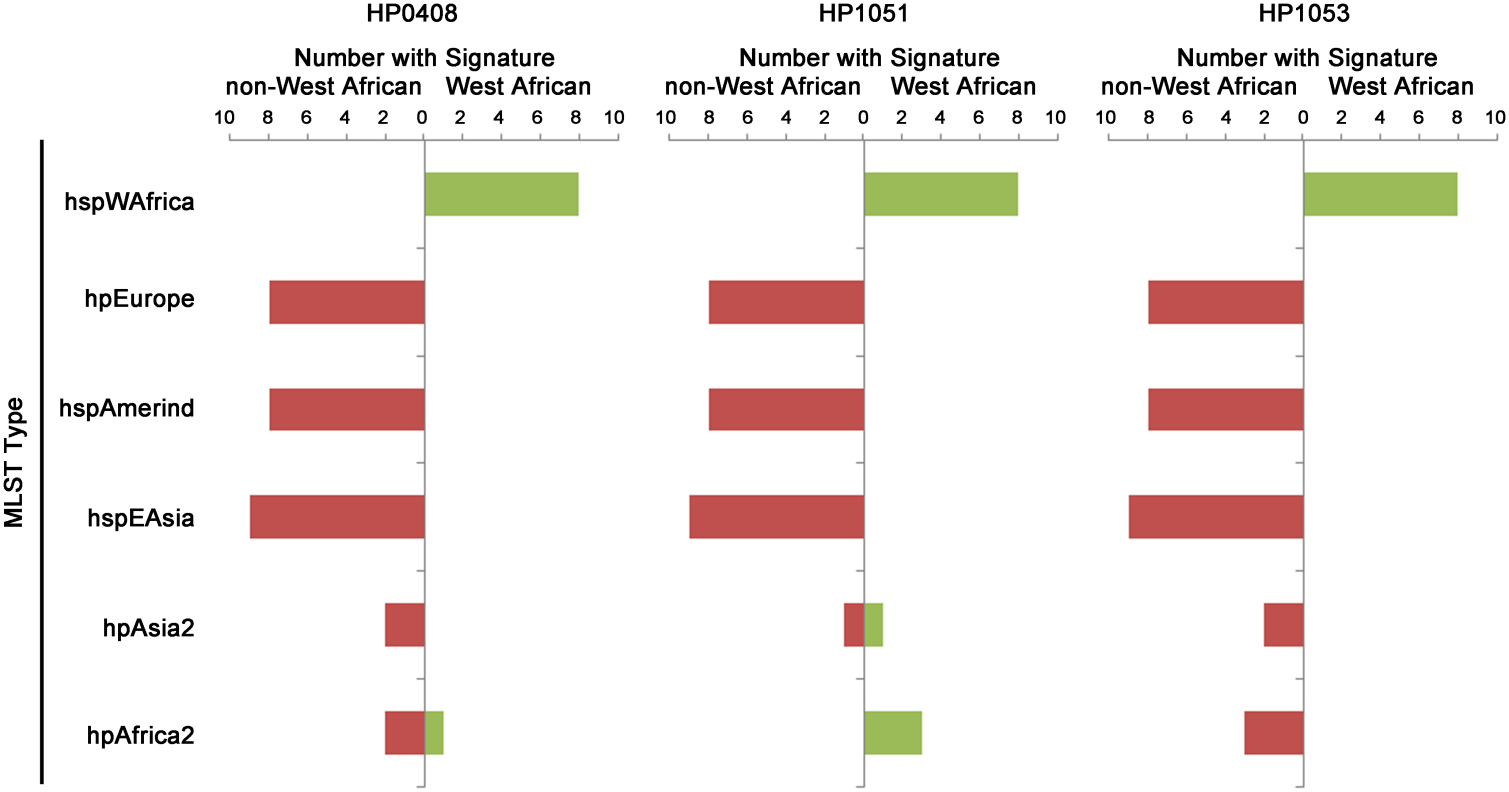
Distribution of West African signatures in strains of diverse geographic origins. Genome sequences of *H*. *pylori* strains previously classified by MLST into the indicated population groups [5, 28, 43] were searched by BLAST to detect the insertions and deletions illustrated in Fig 1. The figure illustrates the number of strains in each population group that contained the insertions or deletions characteristic of hspWAfrica strains.

We also examined genomes from a larger group of strains that were isolated in multiple global locations and previously classified into distinct population groups based on MLST analysis (S5 Table). This analysis confirmed that the insertions and deletions characteristic of hspWAfrica strains (in HP0408, HP0151, and HP0153, see Fig 1) were rare in non-African populations of strains (Fig 3). Collectively, these results indicate that these insertions or deletions are markers for *H*. *pylori* strains of African origin.

## Discussion

*H*. *pylori* exhibits a high level of genetic diversity [1–4], and *H*. *pylori*-associated disease states [especially gastric cancer] exhibit geographic variation in incidence. For example, the incidence of gastric cancer is very high in East Asia, but relatively low in Africa [18, 19]. Therefore, there is considerable interest in the hypothesis that there are geographic differences in *H*. *pylori* virulence. MLST analysis of housekeeping genes is a useful approach for distinguishing among various geographic populations of *H*. *pylori* [5, 6], but the sequence polymorphisms in *H*. *pylori* housekeeping genes are typically synonymous substitutions that are unlikely to be associated with alterations in protein function or differences in bacterial virulence.

In this study, we first analyzed representative strains from seven different *H*. *pylori* populations or subpopulations to identify proteins that exhibit a high level of sequence diversity, a characteristic of rapidly evolving proteins. This analysis revealed a set of 72 such proteins. In contrast, the sequences of many other *H*. *pylori* proteins are highly conserved in this group of strains (S2 Table). The sequences of some of these proteins are known to have distinctive features in specific geographic populations of strains [37–39, 44–51]. For example, one previous study analyzed differences between East Asian *H*. *pylori* strains and non-East Asian strains (hpEurope or hspWAfrica), and identified about 50 proteins that exhibited high levels of sequence diversity [37]. There is considerable overlap between that group of proteins and the set of rapidly evolving proteins identified in the analysis of 7 globally distributed strains in the current study. The high level of sequence divergence detected in a subset of *H*. *pylori* proteins probably reflects the cumulative result of numerous positive selection events [37]. The observed sequence divergence in some proteins could potentially have resulted from horizontal transfer events in which *H*. *pylori* acquired DNA from a closely related species.

We then conducted a more focused analysis to identify a specific group of rapidly evolving proteins, namely, those that have distinctive features in West African strains. This analysis revealed eight proteins that were highly divergent in hspWAfrica strains compared to hpEurope strains. Three of the eight proteins contained amino acid insertions or deletions that were differentially distributed between the two populations. These insertions or deletions, up to 8 amino acids in length, presumably reflect low probability mutational events that occurred rarely during the evolution of *H*. *pylori*. The genes for two of these eight proteins were determined to be under diversifying selection, based on use of the McDonald-Kreitman test, which supports the hypothesis that the divergence of these sequences is the consequence of evolutionary selective pressures. Analyses of additional *H*. *pylori* strains, classified into multiple populations groups by MLST analysis, indicated that the insertions and deletions present in West African strains were rarely detected in other populations of strains (Fig 3). Therefore, these insertions and deletions are useful biomarkers for strains of West African origin.

Rapidly evolving proteins potentially exhibit alterations in activity or acquire new functions as a consequence of their diversification, as exemplified by different activities exhibited by various forms of *H*. *pylori* CagA or VacA [12, 16, 29–33, 52]. CagA exhibits marked sequence divergence in East Asian strains compared to non-East Asian strains [12, 28, 29], but relatively little evidence of divergence when comparing West African and European strains. Therefore, a geographic specialization of individual *H*. *pylori* proteins may be more readily detectable in some geographic regions than in others. We speculate that specific mutations in rapidly evolving *H*. *pylori* proteins confer selective advantages that are most relevant in strains that colonize humans with specific genetic traits or strains exposed to specific gastric environments, which might help to explain the geographic distribution of the corresponding sequences.

Since genome sequences of *H*. *pylori* strains isolated in multiple West African countries are not currently available, the analysis reported in this study utilized six West African strains isolated from patients in The Gambia (all classified as hspWAfrica by MLST analysis) and two strains isolated from patients in Europe or the United States (also classified as hspWAfrica by MLST analysis). BLAST analyses of *H*. *pylori* genomes available in Genbank confirms that these insertions or deletions are present in many *H*. *pylori* strains isolated in The Gambia, in addition to the six Gambian strains analyzed in the current study (data not shown). It is not known at present whether the patterns of sequence divergence reported in the current study are generally representative of all West African strains, or limited to strains from certain regions of West Africa. In future studies, it will be important to analyze genetic features of strains from additional regions of West Africa.

Elucidating genetic features of African *H*. *pylori* strains is relevant for understanding the relatively low rate of gastric cancer reported in Africa. A previous study identified several strain-specific genes that were present more commonly in African strains than in other populations of strains [53], but in the current study, we did not identify any genes that were present uniquely in African strains or European strains. The geographic differences in protein sequences identified in the current study could potentially be associated with alterations in protein function, which might result in alterations of *H*. *pylori* virulence. In future studies, it will be important to investigate possible functional consequences of the observed sequence variations, and further investigate the distribution of these sequence variations to determine if there is any correlation with disease state.

## Materials and methods

### Selection of *H*. *pylori* genomes for analysis

*H*. *pylori* strains were selected for analysis based on the availability of genome sequences in Genbank. Complete genome sequences were analyzed whenever possible. If complete genomes were not available, incomplete genome sequences with the lowest number of contigs were chosen. In an initial study, we analyzed representative genomes from seven *H*. *pylori* populations or subpopulations (hpEurope, hpAfrica2, hspSAfrica, hspEAsia, hspAmerind, hspWAfrica, hpAsia2), all of which were previously classified by MLST analysis. Further analyses were conducted with multiple *H*. *pylori* strains of European or West African origin (classified as hpEurope or hspWAfrica, based on MLST analysis). These genome sequences were initially selected based on published MLST data or based on the geographic location where the strains were isolated (Europe or West Africa). Six of the 8 strains of West African origin were originally isolated in The Gambia, and previous MLST analyses showed that most strains isolated in the Gambia are classified as hspWAfrica, based on MLST analysis [54].

Accession numbers for the genomes analyzed in this study are as follows: 26695 (NC_000915.1), B8 (NC_014256.1), G27 (NC_011333.1), HPAG1 (NC_008086.1), P12 (NC_011498.1), B38 (NC_012973.1), Lithuania75(NC_017362.1), SJM180 (NC_014560.1), J99 (NC_000921.1), 908 (NC_017357.1), Gambia94/24 (NC_017371.1), GAM115Ai (NZ_APDB00000000.1), GAM201Ai (NZ_APDC00000000.1), GAM246Ai (NZ_APDM00000000.1), GAM252T (NZ_APDR00000000.1), GAM260Bsi (NZ_APDV00000000.1), SouthAfrica7 (NC_017361.1), Shi470 (NC_010698.2), India7 (NC_017372.1), F30 (NC_017365.1), and CC33C (NZ_CP011484.1)

### Multi-locus sequence analysis

Multilocus sequence typing was performed on the strains of West African and European origin as described previously [37, 55, 56]. Nucleotide sequences of 7 conserved housekeeping genes (*atpA*, *efp*, *mutY*, *ppa*, *trpC*, *yphC*, and *ureI*) from each strain were extracted from Genbank or an *H*. *pylori* MLST database (http://pubmlst.org/helicobacter), and were concatenated and aligned to corresponding loci from 178 reference strains (previously assigned to *H*. *pylori* populations or subpopulations) using the Muscle algorithm within MEGA7. Phylogenetic relationships were analyzed using MEGA7 [57] with the Kimura 2-parameter model of nucleotide substitution and 1,000 bootstrap replicates.

### Identification of highly divergent protein sequences

Seven representative strains from diverse geographic origins (Table 1) were compared at the whole-genome level using nWayComp, which compares deduced protein sequences and searches for sequence homologies among multiple strains [37, 58]. For each protein encoded by all seven strains, a 7x7 table of amino acid sequence identities was generated, and mean percent amino acid identities were calculated based on all possible comparisons among the 7 strains. The mean ± SD amino acid sequence identity for the full set of 1187 orthologous protein sequences was 94.2 ± 0.06%. We designated a mean percent amino acid identity of <90% as the criterion for highly divergent protein sequences. The gene alignments of divergent genes were examined by eye to exclude possible misalignments or mismatches to known paralogs, and proteins with mean percent amino acid sequence identity values of less than 50% were excluded. The gene numbers of orthologs in reference strains 26695 and J99 were determined using the PyloriGene webserver (http://genolist.pasteur.fr/PyloriGene/).

Eight strains classified as hpEurope and eight strains classified as hspWAfrica based on MLST (S3 Table) were similarly analyzed at the genome-wide level using nWaycomp [58]. For each protein encoded by all 16 strains, a 16x16 table of amino acid sequence identities was generated. Mean percent amino acid identities were calculated based on several comparisons among the 16 strains, and three values were calculated. The first value was the mean percent amino acid sequence identity based on comparisons among only the eight hspWAfrica strains, the second was the mean percent amino acid sequence identity based on comparisons among the eight hpEurope strains, and the third was the mean percent amino acid sequence identity based on comparisons of hspWAfrica strains with hpEurope strains. The African-European result was subtracted from the intra-African result to obtain a first difference value. The African-European result was then subtracted from the intra-European result to obtain a second difference value. If both difference values were >5% (corresponding to >5% difference in amino acid sequence identity), the protein was considered to exhibit a high level of divergence when comparing hspWAfrican and hpEurope strains.

### McDonald-Kreitman test methods

Nucleotide sequences from 16 *H*. *pylori* strains (eight classified as hpEurope and eight classified as hspWAfrica) encoding proteins with divergent sequences were analyzed using the McDonald-Kreitman test [42]. Nucleotide sequences were aligned using Muscle [59]. The McDonald-Kreitman test was performed using an online resource which ignores codons with gaps and applies a Jukes and Cantor divergence correction [60]. *P* indicates the polymorphisms within the populations, and *D* indicates the fixed divergence between populations, with *n* denoting nonsynonymous and *s*, synonymous changes. The Neutrality Index was calculated as NI = (*Pn*/*Ps*)/(*Dn*/*Ds*), and the alpha value depicts the proportion of adaptive substitutions estimated as 1 –NI.

## Supporting information

S1 Table Proteins exhibiting high levels of sequence divergence among strains from seven geographically distributed *H*. *pylori* populations. (XLSX)

S2 Table Examples of proteins exhibiting a high level of sequence conservation when comparing geographically dispersed populations of *H*. *pylori*. (DOCX)

S3 Table Characteristics of strains classified as hpEurope and hspWAfrica. (DOCX)

S4 Table Examples of proteins exhibiting a high level of sequence conservation when comparing hspWAfrica and hpEurope populations of *H*. *pylori*. (DOCX)

S5 Table MLST classification of *H*. *pylori* strains analyzed in this study. (DOCX)

S1 Fig MLST analysis of *H*. *pylori* strains known or predicted to have African or European origins. Neighbor-joining tree constructed using MEGA7 to assign an MLST classification based on concatenated sequences of the seven conserved housekeeping genes (*atpA*, *efp*, *mutY*, *ppa*, *trpC*, *ureA*, and *yphC*). A set of sequences previously assigned to distinct populations or subpopulations are included as references. Eight strains analyzed in the current study were classified as hpEurope (blue circles) and eight were classified as hspWAfrica (red circles).
(TIF)
