# Supplementary material for: Genetic signatures for Helicobacter pylori strains of West African origin
Source: PLoS One. 2017 Nov 29;12(11):e0188804. doi: 10.1371/journal.pone.0188804 (PMC5706691; doi:10.1371/journal.pone.0188804)
Supplement: S2 Table — (DOCX) [file pone.0188804.s002.docx]

S2 Table. Examples of proteins exhibiting a high level of sequence conservation when comparing geographically dispersed populations of *H. pylori*

| Gene number  (strain 26695) | Gene number  (strain J99) | Mean % amino acid identity^a^ | Annotation or predicted function |
| --- | --- | --- | --- |
| HP1152 | JHP1079 | 98.0 | Signal recognition particle protein |
| HP0422 | JHP0962 | 98.7 | Arginine decarboxylase |
| HP1195 | JHP1118 | 99.2 | Translation elongation factor G |
| HP1196 | JHP1119 | 99.3 | 30S ribosomal protein S7 |
| HP1298 | JHP1218 | 99.6 | Translation Initiation factor IF-1 |
| HP1302 | JHP1222 | 99.8 | 30S ribosomal protein S5 |

^a^ Based on comparisons among 7 globally dispersed strains of *H. pylori.*
