# Supplementary material for: Genetic signatures for Helicobacter pylori strains of West African origin
Source: PLoS One. 2017 Nov 29;12(11):e0188804. doi: 10.1371/journal.pone.0188804 (PMC5706691; doi:10.1371/journal.pone.0188804)
Supplement: S3 Table — (DOCX) [file pone.0188804.s003.docx]

S3 Table. Characteristics of strains classified as hpEurope and hspWAfrica

| Strain | MLST classification | Geographic Source | Disease State | *cagA^a^* | Genome size (Mb) | Jhp0153-jhp0152 180-bp insertion^b^ |
| --- | --- | --- | --- | --- | --- | --- |
| 26695 | hpEurope | United Kingdom | Gastritis | + | 1.67 | - |
| B8 | hpEurope | USA | Gastric cancer | + | 1.68 | - |
| G27 | hpEurope | Italy | Not known | + | 1.66 | - |
| HPAG1 | hpEurope | Sweden | Chronic atrophic gastritis | + | 1.61 | - |
| P12 | hpEurope | Germany | Duodenal ulcer | + | 1.68 | - |
| B38 | hpEurope | France | MALT lymphoma | - | 1.58 | - |
| Lithuania75 | hpEurope | Lithuania | Not known | + | 1.64 | - |
| SJM180 | hpEurope | Peru | Gastritis | + | 1.65 | + |
| J99 | hspWAfrica | USA | Duodenal ulcer | + | 1.64 | + |
| 908 | hspWAfrica | France | Duodenal ulcer | + | 1.55 | + |
| Gambia94/24 | hspWAfrica | Gambia | Not known | + | 1.71 | + |
| GAM115Ai | hspWAfrica | Gambia; Greater Banjul Area; Wollof ethnicity | Gastric erosion | + | 1.68 | + |
| GAM201Ai | hspWAfrica | Gambia; Greater Banjul Area; Sarahule ethnicity | Gastritis | - | 1.61 | + |
| GAM246Ai | hspWAfrica | Gambia; Greater Banjul Area; Mandinka ethnicity | Normal endoscopy | + | 1.66 | + |
| GAM252T | hspWAfrica | Gambia; Kiang; Fula ethnicity | Gastric ulcer | + | 1.58 | + |
| GAM260Bsi | hspWAfrica | Gambia; Greater Banjul Area; Wollof ethnicity | Normal endoscopy | + | 1.57 | + |

^a^ Presence or absence of the *cagA* gene in the indicated genome.

^b^ Presence or absence of a 180-bp insertion, located in the intergenic region between JHP0153 and JHP1052 in strain J99, in the indicated genomes.
