# Supplementary material for: Genetic signatures for Helicobacter pylori strains of West African origin
Source: PLoS One. 2017 Nov 29;12(11):e0188804. doi: 10.1371/journal.pone.0188804 (PMC5706691; doi:10.1371/journal.pone.0188804)
Supplement: S4 Table — (DOCX) [file pone.0188804.s004.docx]

S4 Table. Examples of proteins exhibiting a high level of sequence conservation

when comparing hspWAfrica and hpEurope populations of *H. pylori*

| Gene number  (strain 26695) | Mean % amino acid identity, hpEurope-hspWAfrica | Mean % amino acid identity, intra-hpEurope | Mean % amino acid identity, intra-hspWAfrica | Annotation or predicted function |
| --- | --- | --- | --- | --- |
| HP1152 | 98.3 | 98.6 | 98.8 | Signal recognition particle protein |
| HP0422 | 99.2 | 99.2 | 99.5 | Arginine decarboxylase |
| HP1195 | 99.2 | 99.3 | 99.6 | Translation elongation factor G |
| HP1196 | 99.2 | 99.5 | 99.9 | 30S ribosomal protein S7 |
| HP1298 | 99.7 | 99.3 | 100 | Translation Initiation factor IF-1 |
| HP1302 | 99.7 | 99.7 | 99.7 | 30S ribosomal protein S5 |
