# Supplementary material for: Genetic signatures for Helicobacter pylori strains of West African origin
Source: PLoS One. 2017 Nov 29;12(11):e0188804. doi: 10.1371/journal.pone.0188804 (PMC5706691; doi:10.1371/journal.pone.0188804)
Supplement: S5 Table — (DOCX) [file pone.0188804.s005.docx]

S5 Table: MLST classification of *H. pylori* strains analyzed in this study

Strain MLST classification

26695 hpEurope

G27 hpEurope

P12 hpEurope

B8 hpEurope

HPAG1 hpEurope

B38 hpEurope

Lithuania75 hpEurope

SJM180 hpEurope

J99 hspWAfrica

Gambia94-24 hspWAfrica

GAM115Ai hspWAfrica

GAM201Ai hspWAfrica

GAM246Ai hspWAfrica

GAM252T hspWAfrica

GAM260Bsi hspWAfrica

35A hspEAsia

51 hspEAsia

52 hspEAsia

98-10 hspEAsia

908 hspEAsia

F16 hspEAsia

F30 hspEAsia

F32 hspEAsia

F57 hspEAsia

Cuz20 hspAmerind

Puno120 hspAmerind

Puno135 hspAmerind

Sat464 hspAmerind

Shi112 hspAmerind

Shi169 hspAmerind

Shi417 hspAmerind

Shi470 hspAmerind

v225d hspAmerind

SouthAfrica7 hpAfrica2

SouthAfrica20 hpAfrica2

SouthAfrica50 hpAfrica2

India7 hpAsia2

SNT49 hspAsia2

CC33C hspSAfrica
