# Supplementary figures and images for: Genetic signatures for Helicobacter pylori strains of West African origin
Source: PLoS One. 2017 Nov 29;12(11):e0188804. doi: 10.1371/journal.pone.0188804 (PMC5706691; doi:10.1371/journal.pone.0188804)

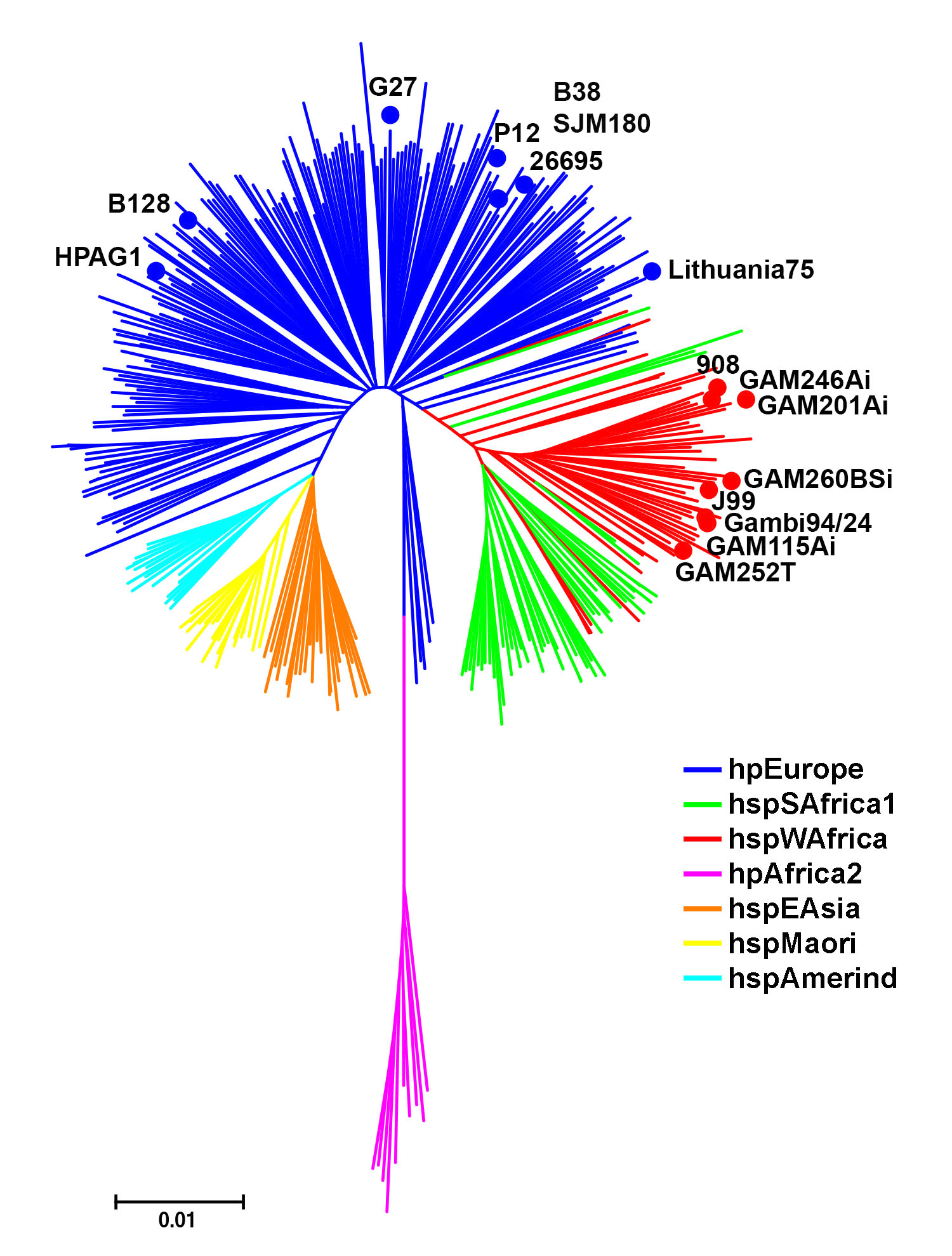

Supplement: S1 Fig — Neighbor-joining tree constructed using MEGA7 to assign an MLST classification based on concatenated sequences of the seven conserved housekeeping genes (atpA, efp, mutY, ppa, trpC, ureA, and yphC). A set of sequences previously assigned to distinct populations or subpopulations are included as references. Eight strains analyzed in the current study were classified as hpEurope (blue circles) and eight were classified as hspWAfrica (red circles). (TIF) [file pone.0188804.s006.tif]
